# East *versus* West: the great divide

**DOI:** 10.1192/bji.2018.13

**Published:** 2019-02

**Authors:** Gunjan Sharma

**Affiliations:** Foundation Year One Doctor, Trauma and Orthopaedics, North Devon District Hospital, Barnstaple, UK. Email gunjan.sharma@nhs.net

The medical elective is one of the jewels of British medical education. For some, it is an opportunity to help the world's poorest people, whereas for others it offers 2 months of freedom. Regardless of our motives, all of us walk into a different culture with our own ideas about how the world should look. The elective is all about shattering those ideas to the ground.

I travelled to India as an idealistic British medical student, under the assumption that inadequate resources were the only barriers to good mental healthcare in the East. This elective, an 8-week period in a psychiatric hospital in rural northern India, gave me an insight into the intricate links between culture, religion, spirituality and mental healthcare. Most importantly, it raised questions on the practice of medicine back in the UK, which can be summarised broadly under three headings: autonomy; ‘ideas, concerns and expectations’; and confidentiality.

## Autonomy

In India, status is an important lubricant of society (Sankaran *et al*, 2017). The medical doctor stands near the top of this hierarchy, with the powers to heal the sick and dying.

The status of the doctor is closely linked with the status of the patient and how they are subsequently treated (Truog, 2012). In India, teachings from Hinduism emphasise healing as an art ‘worthy of men’, leading to a reverence of the medical doctor which, from a Western perspective, can at times feel uncomfortable (Kaba & Sooriakumaran 2007). This is a stark contrast to the patient-centred care we emphasise in the UK, which aims to empower the patient and encourages them to make their own choices (Royal College of General Practitioners, 2014).

Patient expectations in rural India are different. In the psychiatric ward, patients would sit on the edge of their beds, speaking only when spoken to. Families would watch in silence as doctors prescribed medications, fearful of breaching the unwritten rule and questioning an ‘elder’. The paternalistic model could be seen in every aspect of a patient's care, reflecting the deeper links between respect and communication.

The East and the West can stereotypically be divided into individualistic and collectivist societies (Darwish & Huber, 2003). The way we view our society affects the way we practise healthcare. In the UK, doctors treat the individual patient with an emphasis on consultations behind closed doors; whereas in other societies, the professional may be treating the family or even the entire village. Patient-centred care is just another, culturally appropriate way of practicing medicine, with patient autonomy seen as a consequence of individualistic society rather than an objective branch of medical ethics.

## Ideas, concerns and expectations

In the UK we are also taught to focus on the patients' ideas, concerns and expectations, with research illustrating that this focus is linked with improved health outcomes and greater rapport between clinician and patient (Matthys *et al*, 2009). During this elective, the phrase was reversed with the focus being on the ideas, concerns and expectations of the clinician leading the consultation. In almost every conversation, the opinion of the clinician would be adhered to without question. Further discussion with family members after the ward round would unleash a wider range of opinions, but always with the acknowledgement that the doctor knew best. Interestingly, many families voiced the belief that one came to hospital to be told what to do; the role of the patient was to listen and obey. When I raised the idea of a clinician asking what the patient wanted, most families understood this to mean that the doctor did not know what they were doing.

This difference in consultation styles has been linked to a multitude of factors, such as hierarchy and education. Etiquette is an important part of communication and the beliefs that we hold about another individual, particularly one who is perceived to be of higher status, have an impact on what we say and what we do not (Claramita *et al*, 2013). This is of particular relevance when we reflect upon our own consultation practices in the West. We may not live in the paternalistic model, but the doctor–patient relationship builds boundaries that can lead to things being left unsaid, whether it be something as simple as not wishing to impose upon the ‘busy’ doctor's time, or the fear that one will not be taken seriously because of ‘mild’ symptoms.

## Confidentiality

Confidentiality in Western healthcare is one of the pinnacles of good medical practice (General Medical Council, 2017). The right to privacy and the ability to live our lives as we wish are principles that are embedded in European Law (European Convention on Human Rights, 2003). This is in stark contrast to a society in which one is a cog in the wheel; the individual carries the beliefs and values of not only their family, but the norms of their society and the etiquette of their culture (Hui & Triandis, 1986). In such a world, confidentiality takes on a whole new meaning.

My first ward round in India was a powerful illustration of this difference. Ten family members surrounded the first patient, an elderly gentleman who lay in silence, gazing at the ceiling. Each one of these ten relatives was involved in this patient's care. Every neighbour in the local area knew that this farmer had been forced into a psychiatric hospital, physically restrained and sedated. They knew that he had tried to hit his wife several times, that he had smashed the furniture and screamed into the night. The entire village knew what was happening to this gentleman every day that he was in hospital.

When patients were first admitted into hospital, it was always the family member who was drawn into the conversation about the diagnosis and management. Discussions would be held with families about how their loved one should be cared for at home, the importance of exercise and regular meals, as if they were talking about someone who was not there. The ward would be filled during visiting times, neighbours and shopkeepers all coming to give their condolences and to hear about what was happening to the young boy from down the road. Patients’ stories were not just documented on the ward round or discussed in meetings; they were gossiped about in the local cafe, sniggered at by young children and disapproved of by the elders. Being psychologically unwell was not a personal journey but a theatre.

## Conclusion

This brief elective in India illustrates the diversity of medical practice. One can become so ingrained in the teachings of British medicine, with its emphasis on patient autonomy and healthcare as a public service, that the elective can raise feelings of tension and unease.

But the elective is also a powerful tool that can help us appreciate the symbiotic relationship between medicine, culture and the patient. There is no ‘right’ or ‘wrong’ way to practice medicine, but reflecting on our own practice can make us aware of our own expectations of how patients should present and how society should treat the unwell. In an age of globalisation and multiculturalism, the ability to acknowledge such bias in ourselves will make us more sensitive to our own Western values and ensure that they do not overwhelm our patient–doctor relationship.
